# Dehydroepiandrosterone Protects Endothelial Cells against Inflammatory Events Induced by Urban Particulate Matter and Titanium Dioxide Nanoparticles

**DOI:** 10.1155/2013/382058

**Published:** 2013-01-14

**Authors:** Elizabeth Huerta-García, Angélica Montiél-Dávalos, Ernesto Alfaro-Moreno, Gisela Gutiérrez-Iglesias, Rebeca López-Marure

**Affiliations:** ^1^Departamento de Biología Celular, Instituto Nacional de Cardiología “Ignacio Chávez”, Juan Badiano No. 1, Colonia Sección 16, Tlalpan, 14080 México, DF, Mexico; ^2^Departamento de Posgrado. Escuela Superior de Medicina, Instituto Politécnico Nacional, Mexico; ^3^Subdirección de Investigación Básica, Instituto Nacional de Cancerología, Mexico

## Abstract

Particulate matter (PM) and nanoparticles (NPs) induce activation and dysfunction of endothelial cells characterized by inhibition of proliferation, increase of adhesion and adhesion molecules expression, increase of ROS production, and death. DHEA has shown anti-inflammatory and antioxidant properties in HUVEC activated with proinflammatory agents. We evaluated if DHEA could protect against some inflammatory events produced by PM_10_ and TiO_2_ NPs in HUVEC. Adhesion was evaluated by a coculture with U937 cells, proliferation by crystal violet staining, and oxidative stress through DCFDA and Griess reagent. PM_10_ and TiO_2_ NPs induced adhesion and oxidative stress and inhibited proliferation of HUVEC; however, when particles were added in combination with DHEA, the effects previously observed were abolished independently from the tested concentrations and the time of addition of DHEA to the cultures. These results indicate that DHEA exerts significant anti-inflammatory and antioxidative effects on the damage induced by particles in HUVEC, suggesting that DHEA could be useful to counteract the harmful effects and inflammatory diseases induced by PM and NPs.

## 1. Introduction

Particulate matter (PM) is an environmental factor that has been associated with increased cardiovascular morbidity and mortality, particularly mass concentrations of PM with aerodynamic sizes ≤2.5 or ≤10 **μ**M (PM_2.5_, PM_10_). Numerous studies have shown associations between PM and risk of cardiac ischemia and arrhythmias, increased blood pressure, decreased heart rate variability, and increased circulating markers of inflammation and thrombosis [1]. Also, ultrafine particles (UFPs; PM < 0.1 **μ**M) induce oxidative stress leading to inflammation and resulting in respiratory and cardiovascular disease, because they have high pulmonary deposition efficiency and their magnitudes in the particle number concentration are higher than larger particles; thus they have a much larger surface area. Such is the case of titanium dioxide nanoparticles (TiO_2_ NPs) that cause several adverse effects on mammalian cells such as increase of reactive oxygen species (ROS) production and cytokines levels, reduction of cell viability and proliferation, and induction of apoptosis and genotoxicity [2].

We have previously shown that PM_2.5_ and PM_10_ induce adhesion of U937 cells to human umbilical vein endothelial cells (HUVEC), which was associated with an increase in the expression of adhesion molecules such as E- and P-selectins, ICAM-1, PECAM-1, and VCAM-1 [3, 4]; besides, they induce production of ROS and NO and nuclear translocation of NF-*κ*B [5]. Also, we have shown that TiO_2_ NPs are internalized into HUVEC; they inhibit strongly cell proliferation; and induced cellular death (necrosis and apoptosis) [6]. Besides, TiO*₂* NPs induce activation of HUVEC through an increase in adhesion and in the expression of adhesion molecules and other molecules involved in the inflammatory process. These effects were associated with oxidative stress and NF-*κ*B pathway activation [6]. Together, all these results indicate that all these particles induce HUVEC activation, suggesting that they may participate in the development of inflammatory diseases.

In previous works, we have shown that dehydroepiandrosterone (DHEA), an adrenal hormone, has shown anti-inflammatory and antioxidative roles in HUVEC treated with two proinflammatory molecules such as TNF-*α* and oxLDL [7, 8]. DHEA decreases the adhesion of monocytic cells to HUVEC, decreases the expression of early and late molecules of adhesion, and interferes with the translocation of NF-*κ*B and I*κ*B-*α* degradation. Also, DHEA inhibits ROS and NO production.

In this work, we hypothesized that DHEA could protect HUVEC against inflammatory events induced by PM_10_ and TiO_2_ NPs. To test this, we exposed HUVEC to PM_10_ and TiO_2_ NPs in combination with DHEA and evaluated the adhesion of monocytic cells, proliferation, and ROS and NO production. 

## 2. Materials and Methods

### 2.1. Materials

RPMI 1640 and M199 media and trypsin were purchased from GIBCO/BRL (Grand Island, NY, USA), and fetal bovine serum (FBS) was HyClone (Logan, UT, USA). Sterile plastic material for tissue culture was from NUNC and COSTAR. Flow cytometry reagents were purchased from Becton Dickinson, Immunocytometry Systems (San José, CA, USA). TNF-*α* was purchased from R & D Systems (Minneapolis, MN, USA). Peroxidase-labeled monoclonal antibody against Von Willebrand factor was purchased from Santa Cruz Biotechnology (Santa Cruz, CA, USA). H_2_DCFDA was purchased from Molecular Probes and TiO_2_ NPs from Paris Drugstore (Mexico City, Mexico). All other chemicals were purchased from Sigma Aldrich (St. Louis, MO, USA). 

### 2.2. Particles and Preparation

PM_10_ were collected from the north zone of Mexico City. Samples were taken three days per week throughout 2007 using a GMW high-volume particle collector (model 1200 VFC HV PM10, Sierra Andersen) to collect particles with mean aerodynamic diameters equal to or smaller than 10 *μ*M. Particles were recovered from the filters as previously described [9].

At least 1 mg of particles was weighed and sterilized by autoclave the night before of each experiment. PM_10_ and TiO_2_ NPs suspensions in M199 medium, at a concentration of 1 mg/mL, were prepared few minutes before cell exposure. Aliquots were taken from these suspensions and further diluted with culture medium until the required final concentration was obtained. TiO_2_ NPs used were previously characterized by our work group [6]. Their characterization showed aggregates of spheres of less than 50 nm with a size distribution of aggregates between 105 and 1281 nm and a mean size of 421 nm, when TiO_2_ NPs were suspended in M199 medium plus 10% FBS. In our assays, NPs were not sonicated because in our previous studies we did not observe difference in the biological effects induced by sonicated or nonsonicated TiO_2_ NPs.

### 2.3. Endothelial Cell Cultures

Primary HUVEC cultures were obtained by proteolytic dissociation of the umbilical cord veins from normal deliveries, treated with collagenase type II (0.2 mg/mL), and cultured on gelatin-coated culture dishes in M199 supplemented with 10% FBS, glutamine (2 mM), heparin (1 mg/mL), and endothelial mitogen (20 *μ*g/mL), as previously described [5]. Cells were used for all experiments on their second passage. The phenotype of HUVEC cultures was confirmed by Von Willebrand antigen staining. Cultures exposed to human recombinant TNF-*α* (10 ng/mL) or H_2_O_2_ (500 *μ*M) were used as positive controls of endothelial activation. 

### 2.4. Culture of U937 Cells

Human leukemia promonocytic U937 cells were cultured in RPMI-1640 medium supplemented with 10% FBS and L-glutamine (2 mM).

### 2.5. Adhesion of U937 Cells to Endothelial Cells

Adhesion was evaluated using U937 cells that were labeled with [^3^H]-thymidine; 1 × 10^5^ HUVEC were seeded in 24-well tissue-culture plates with 1 mL of supplemented M199 medium and treated with TNF-*α* (10 ng/mL), DHEA (1, 10, and 100 *μ*M), TiO_2_ NPs (10 *μ*g/cm^2^), and PM_10_ (20 *μ*g/cm^2^) for different times, whereas 6 × 10^6^ U937 cells were incubated with 30 *μ*Ci of [^3^H]-thymidine for 48 h. Pretreated HUVEC were co-cultivated for 3 h with 5 × 10^5^ U937 cells/well. Each well was washed to eliminate U937 cells not attached to HUVEC. After this, cells were fixed with 95% methanol and lysed with NaOH (200 mM) for 12 h, and radioactivity was determined in a scintillation counter (Beckman Coulter model LS6500, Miami, FL, USA). Counts per minute (cpm) were considered directly proportional to the number of U937 cells adhered to HUVEC.

### 2.6. Crystal Violet Staining

Cell number was evaluated by crystal violet staining. HUVEC were cultured on 96-multiwell plates without and with DHEA (1, 10, and 100 *μ*M), TiO_2_ NPs (10 *μ*g/cm^2^), and PM_10_ (20 *μ*g/cm^2^) for 72 h. DHEA was added 1 h before exposure to particles. At the end of these treatments, cells were fixed with 100 *μ*L of ice cold glutaraldehyde (1.1% in PBS) for 15 min at 4°C. Plates were washed three times by submersion in deionized water, air-dried, stained for 20 min with 100 *μ*L of a 0.1% crystal violet solution (in 200 mM phosphoric acid buffer at pH 6). After careful aspiration of the crystal violet solution, the plates were extensively washed with deionized water, air-dried prior to the solubilization of the bound dye with 100 *μ*L of a 10% acetic acid solution, and incubated during 30 min. Optical density of the plates was measured at 595 nm in a multiplate spectrophotometer.

### 2.7. Measurement of Reactive Oxygen Species

The oxidation of 2,7-dichlorodihydrofluorescein diacetate (H_2_DCFDA) into 2,7-dichlorodihydrofluorescein (DCF) was used to assess Ros generation. HUVEC were cultured without or with DHEA (1, 10, and 100 *μ*M), TiO_2_ NPs (10 *μ*g/cm^2^), and PM_10_ (20 *μ*g/cm^2^) or in combination for 3 h. DHEA was added 1 h before particles. H_2_O_2_ (500 *μ*M) was used as positive control to induce oxidative stress. After treatment, cells were incubated with H_2_DCFDA (10 *μ*M) for 30 min at 37°C and washed twice with PBS. After an extensive wash, fluorescence was evaluated by flow cytometry (Facscalibur, Becton Dickinson). The mean fluorescence intensity was calculated by multiplying the number of events (fluorescent cells) by the mean of the intensity presented by the Cell Quest software used for the analysis.

### 2.8. Production of NO

Quantification of nitrite was used as an indirect method to determine the production of NO. Cells were seeded in 96 well plates (NUNC) at a density of 1 × 10^5^ cells/well in M199 (phenol red free) and 10% FBS. Cells were cultured without or with DHEA (1, 10, and 100 *μ*M), TiO_2_ NPs (10 *μ*g/cm^2^), and PM_10_ (20 *μ*g/cm^2^) or in combination for 72 h. DHEA was added 1 h before particles. Unexposed cultures were used as negative controls. After treatment, 100 *μ*L of the conditioned medium was diluted 1 : 2 with 100 *μ*L of Griess solution and incubated for 15 min at room temperature. Previously, a standard curve was performed using known concentrations of NaNO_2_. The optical density of the plates was measured at 540 nm (Microplate autoreader EL311, Bio-Tek Instruments, Winooski, VT, USA). The concentrations of NaNO_2_ in control and exposed cultures were plotted against the standard.

### 2.9. Statistical Analysis

All the endpoints were measured at least three times. The results are expressed as mean ± standard deviation. Statistical significance was evaluated using one-way analysis of variance (ANOVA) test using GraphPad Prism, version 2.0 (GraphPad Software, CA, USA), followed by Duncan's multiple range test (MRT), to assess differences between group means. Differences were considered significant when *P* < 0.01. When a temporal curve was used to evaluate the nitrite production, the exposed cultures were compared with the controls at the respective time point.

## 3. Results

### 3.1. DHEA Inhibited the Adhesion Induced by TiO_2_ NPs and PM_10_

**Figure 1:**
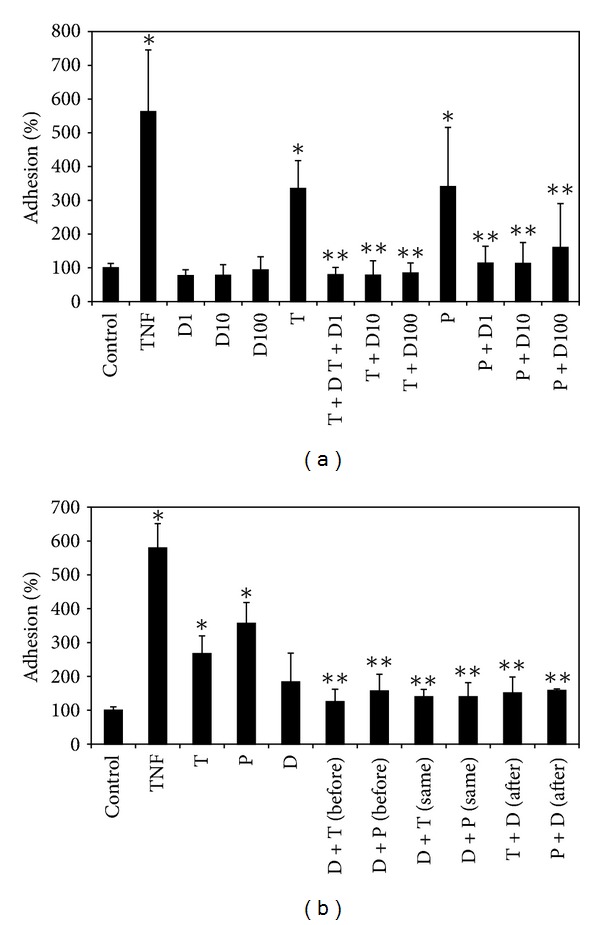
Effect of DHEA on the adhesion induced by particles. Cells were treated with 1 (D1), 10 (D10), and 100 *μ*M (D100) of DHEA alone or in combination with 10 *μ*g/cm^2^ of TiO_2_ NPs (T) or 20 *μ*g/cm^2^ of PM_10_ (P) for 24 h (a). After this, U937 cells labeled with [^3^H]-thymidine were cultured with HUVEC for 3 h more, and adhesion was evaluated in a scintillation counter. TNF-*α* (10 ng/mL) was used as a positive control. In (b), DHEA was added 1 h before (before), at the same time (same), and 1 h after (after) the addition of TiO_2_ NPs or PM_10_. The results were expressed as percentage of adhesion with respect to untreated cells (100%) and shown as mean ± SD of three separate experiments. *P < 0.01 compared with nontreated cells, and **P < 0.01 compared with particles-treated cells.

Adhesion of U937 cells to HUVEC was evaluated by a coculture assay. DHEA alone did not induce adhesion, whereas the treatment with TiO_2_ NPs and PM_10_ induced a 2-fold increase in adhesion, compared to untreated cells; however, this was significantly inhibited until reaching basal levels when HUVEC were exposed to a pretreatment with DHEA (Figure 1(a)). All concentrations of DHEA inhibited the increase of adhesion induced by particles. In order to determine if the time of addition of DHEA was important to exert its protective effect, DHEA was added to HUVEC before, at the same time, and after treatment with TiO_2_ NPs and PM_10_. DHEA inhibited the adhesion induced by the particles independently from the time of addition (Figure 1(b)). 

### 3.2. DHEA Abolished the Decrease of Proliferation Induced by TiO_2_ NPs and PM_10_

**Figure 2:**
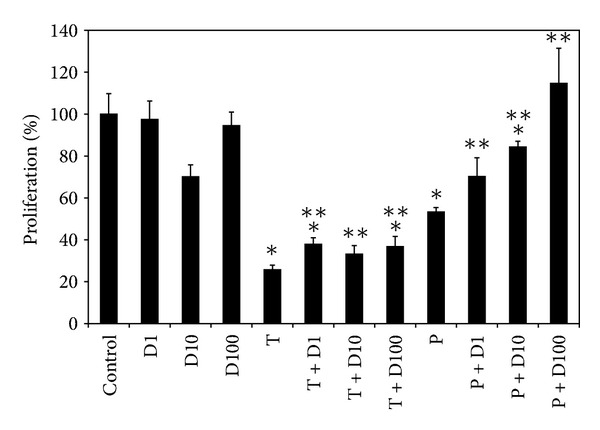
Effect of DHEA on the inhibition of proliferation induced by particles. Cells were treated with 1 (D1), 10 (D10), and 100 *μ*M (D100) of DHEA alone or in combination with 10 *μ*g/cm^2^ of TiO_2_ NPs (T) or 20 *μ*g/cm^2^ of PM_10_ (P) for 72 h. Cell proliferation was evaluated by crystal violet staining. Nontreated cells showed 100% of proliferation. The results are expressed as mean ± SD of three separate experiments. *P < 0.01 compared with nontreated cells, and **P < 0.01 compared with particles-treated cells.

To examine the possible involvement of DHEA on the inhibition of proliferation induced by TiO_2_ NPs and PM_10_, HUVEC were exposed to DHEA alone or in combination with the particles, and proliferation was evaluated by crystal violet. Results showed that DHEA reverted almost completely the inhibition of proliferation induced by TiO_2_ NPs at any concentration (Figure 2); however, DHEA at 100 *μ*M in combination with PM_10_ (P + D100) abolished 100% the inhibition induced by PM_10_ alone. 

### 3.3. DHEA Abolished the Increase of ROS and NO Induced by TiO_2_ NPs and PM_10_

**Figure 3:**
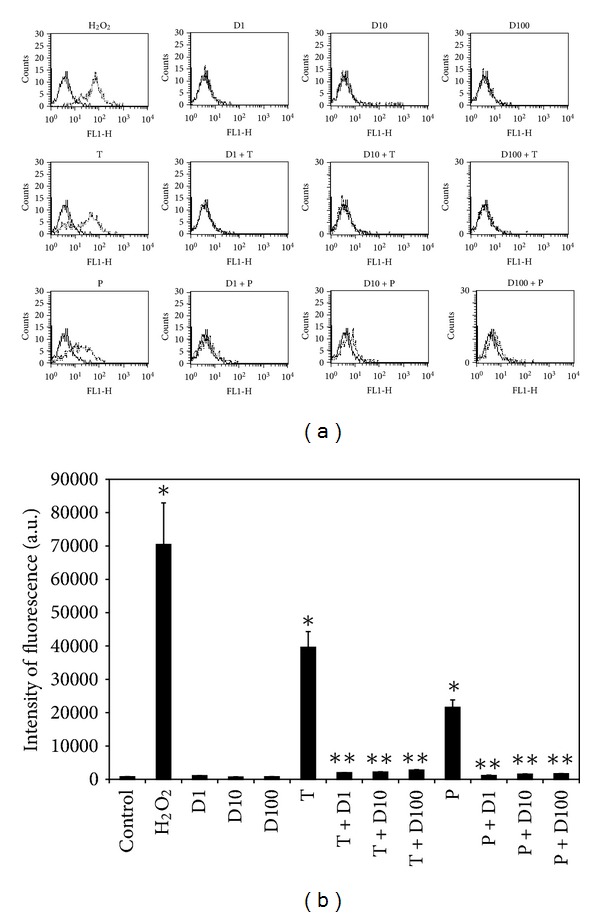
Effect of DHEA on ROS production induced by particles. Cells were treated with 1 (D1), 10 (D10), and 100 *μ*M (D100) of DHEA alone or in combination with 10 *μ*g/cm^2^ of TiO_2_ NPs (T) or PM_10_ (P) for 48 h. H_2_O_2_ (500 *μ*M) was used as a positive control. ROS concentration was evaluated using H_2_DCFDA by flow cytometry. In (a), continuous lines correspond to control cells without treatment, and dashed lines correspond to treated cells. Histograms match to one representative experiment of three performed in an independent way. In (b), fluorescence intensity was calculated through multiplying the number of events by the mean of the fluorescence intensity value. The results are expressed as mean ± SD of three separate experiments. *P < 0.01 compared with nontreated cells, and **P < 0.01 compared with particles-treated cells.

**Figure 4:**
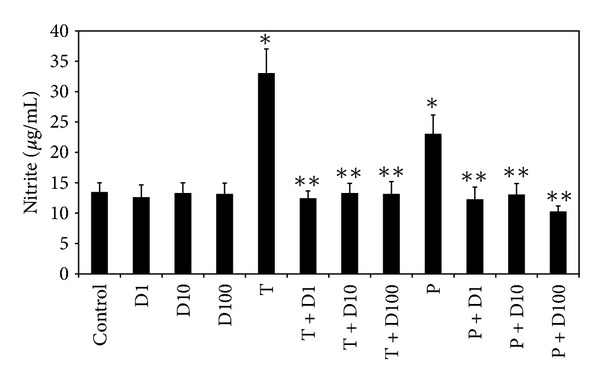
Effect of DHEA on NO production induced by particles. Cells were treated with 1 (D1), 10 (D10), and 100 *μ*M (D100) of DHEA alone or in combination with 10 *μ*g/cm^2^ of TiO_2_ NPs (T) or PM_10_ (P) for 72 h. NO concentration was evaluated using Griess reagent. Previously, a standard curve was performed using known concentrations of nitrite. Absorbance of the concentrations of control and problem samples was plotted against the standard curve. Data are represented as concentration of nitrite (*μ*g/mL) and are expressed as mean ± SD of three separate experiments. * Indicates *P* < 0.01 compared with control cells, and **P < 0.01 compared with particles-treated cells.

Oxidative stress was determined indirectly by measuring the H_2_O_2_ and nitrite production by H_2_DCFDA and Griess reagent, respectively. After exposure to TiO_2_ NPs and PM_10_ for 24 h, fluorescence from most cells stained with H_2_DCFDA indicated that intracellular H_2_O_2_ had accumulated strongly in HUVEC; however, this was significantly inhibited reaching almost basal levels by pretreatment with DHEA at all concentrations used (Figure 3). In relation to NO production, TiO_2_ NPs and PM_10_ induced approximately an increase of 150% and 70% of nitrite concentration, respectively. When DHEA was added in combination with any of the particles, the induction was completely abolished to control levels (Figure 4).

## 4. Discussion

Our previous study showed that exposure of human endothelial cells to TiO_2_ NPs and PM_10_ caused cytotoxic damage [7, 8]. We also have observed that DHEA has an anti-inflammatory and antioxidant effect, protecting HUVEC against the damage induced by TNF-*α* and oxLDL [1, 2]. In the present work, we determined that DHEA protects HUVEC against some inflammatory and oxidative effects induced by PM and NPs.

DHEA, at different concentrations, inhibited the adhesion of U937 cells to HUVEC induced by TiO_2_ NPs and PM_10_, independently from the time of administration of DHEA to the culture (Figure 1). Similar results have been found by Curatola and collaborators [10]. They observed that DHEA inhibited the adhesion of monocytes to cultured human coronary artery endothelial cells (HCAEC), in an estrogen- and androgen-receptor-dependent manner. Besides, DHEA is able to abolish the adhesion of U937 cells to HUVEC treated with proinflammatory molecules such as TNF-*α* and oxLDL and high concentrations of glucose [1, 2, 11]. 

In addition, we observed that the antiproliferative effect induced by TiO_2_ NPs and PM_10_ on HUVEC was similarly reverted with DHEA (Figure 2). It has been described that the toxic potential of NPs is stronger than that induced by PM, because NPs have a much larger surface area, resulting in a high reactivity [12]; nevertheless, we showed that DHEA inhibited the antiproliferative effect of both particles, independently from their size. 

DHEA, at all tested concentrations, abolished completely the oxidative stress induced by TiO_2_ NPs and PM_10_, decreasing the H_2_O_2_ and nitrite production (Figures 3 and 4). Some works have reported that the antioxidant effect of DHEA depends on its concentration [13, 14]. When DHEA was used at physiological concentrations in Chang liver cells, a protection against lipid peroxidation and cell death induced by cumene was observed; but in contrast, at pharmacological concentrations (10–50 *μ*M), DHEA increased both lipid peroxidation and cell death after the prooxidant stimulus [15]. In the present study, we found that, at concentrations ranging from 1 to 100 *μ*M, DHEA exerted an antioxidant effect. In contrast, other anti-inflammatory steroids such as dexamethasone induce oxidative stress [16]. Some works have shown that glucocorticoids therapy can elicit a variety of symptoms and signs, including growth retardation in children; immunosuppression; cardiovascular disorders like hypertension and atherosclerosis; osteoporosis; myopathy; and diabetes mellitus [17], while most importantly, no significant adverse or negative side effects of DHEA have been reported in clinical studies of men and women [18]. 

In other cells, it has been described that DHEA prevented the increased death evoked by glucose deprivation by inhibiting the production of superoxide anion in immunostimulated C6 glioma cells [19] and attenuated lipid peroxidation in high-glucose cultured mesangial cells [20]. In endothelial cells, we previously showed that DHEA inhibits ROS and NO production induced by high concentrations of glucose [11].

As well, in an *in vivo* model using ovariectomized rats, DHEA treatment restored the reduced Cu/Zn-SOD protein expression and eNOS phosphorylation and the increased NADPH oxidase protein expression in the aorta [21]. In rabbits fed with a high-fat diet supplemented with low-dose of DHEA, it showed a partial reduction of oxidative stress restoring the oxidative balance and the inflammatory state, showing a beneficial effect [22]. Besides, pretreatment with sulfated DHEA (DHEAS) reverses the stress-induced changes in behavioral and oxidative stress markers and also brain NOx levels in rats [23]. In healthy male Wistar rats, DHEA exerted a protective effect, particularly in the colon, by reducing the tissue susceptibility to oxidation of both lipids and proteins [24]. As a whole, these results suggest an important action of DHEA, improving endothelial function and having a beneficial action by acting as an antioxidant, when cells are exposed to several inflammatory molecules such as TNF-*α*and oxLDL, high concentrations of glucose, and particles. All these results suggest that anti-inflammatory effects induced by DHEA share a similar signaling pathway.

In conclusion, our results show that DHEA could be useful as a protective agent in the prevention and treatment of inflammatory and cardiovascular effects induced by urban particulate matter and nanoparticles where endothelial dysfunction is involved.

## Abbreviations

DHEA: Dehydroepiandrosterone
TNF-*α*: Tumor necrosis factor alpha
HUVEC: Human umbilical vein endothelial cells
ROS: Reactive oxygen species
H_2_DCFDA: 2,7-Dichlorodihydrofluorescein diacetate.
